# Supplementary material for: Comparison of risks of arterial thromboembolic events and glaucoma with ranibizumab and aflibercept intravitreous injection: A nationwide population‐based cohort study
Source: PLoS One. 2022 Apr 18;17(4):e0267088. doi: 10.1371/journal.pone.0267088 (PMC9015139; doi:10.1371/journal.pone.0267088)
Supplement: S1 Table — (DOCX) [file pone.0267088.s001.docx]

| S1 Table. ICD-9/10-CM and ATC code for drug | | |
| --- | --- | --- |
| Variable | **ICD-9-CM / ATC code** | **ICD-10** |
| nAMD | 362.52, 362.53, 362.42, 362.43 | H3532, H35351, H35352, H35353, H35359, H35721, H35722, H35723, H35729, H35731, H35732, H35733, H35739 |
| Diabetes with ophthalmic manifestations | 250.5, 249.5, 362.0 | E08311, E08319, E08321, E08329, E08331, E08339, E08341, E08349, E08351, E08359, E0836, E0839, E09311, E09319, E09321, E09329, E09331, E09339, E09341, E09349, E09351, E09359, E0936, E0939, E10311, E10319, E1036, E1039, E1065, E11311, E11319, E11321, E11329, E11331, E11339, E11341, E11349, E11351, E11359, E1136, E1139, E1165, E13311, E13319, E13321, E13329, E13331, E13339, E13341, E13349, E13351, E13359, E1336, E1339 |
| RVO | 362.35  365.36 | H34811, H34812, H34813, H34819  H34831, H34832,H34833,H34839 |
| Hypertension | 401-405 | I10, I110, I119, I120, I129, I130, I1310, I1311, I132, I150, I151, I152, I158, I159, N262 |
| Diabetes | 250 | E0800, E0801, E08311, E08319, E08321, E08329, E08331, E08339, E08341, E08349, E08351, E08359, E0836, E0839, E0840, E0841, E0842, E0843, E0844, E0849, E0851, E0852, E0859, E08641, E088, E0900, E0901, E09311, E09319, E09321, E09329, E09331, E09339, E09341, E09349, E09351, E09359, E0936, E0939, E0940, E0941, E0942, E0943, E0944, E0949, E0951, E0952, E0959, E09641, E098, E1010, E1011, E1021, E1022, E1029, E10311, E10319, E1036, E1039, E1040, E1041, E1044, E1049, E1051, E1052, E1059, E10610, E10618, E10620, E10621, E10622, E10628, E10630, E10638, E10641, E10649, E1065, E1069, E108, E109, E1100, E1101, E1121, E1122, E1129, E11311, E11319, E11321, E11329, E11331, E11339, E11341, E11349, E11351, E11359, E1136, E1139, E1140, E1141, E1142, E1143, E1144, E1149, E1151, E1152, E1159, E11610, E11618, E11620, E11621, E11622, E11628, E11630, E11638, E11641, E11649, E1165, E1169, E118, E119, E1300, E1301, E1311, E1321, E1322, E1329, E13311, E13319, E13321, E13329, E13331, E13339, E13341, E13349, E13351, E13359, E1336, E1339, E1340, E1341, E1342, E1343, E1344, E1349, E1351, E1352, E1359, E13641, E138, E139 |
| Renal disease | 585, 586, 403, 404 | I120, I129, I130, I1310, I1311, I132, N184, N185, N186, N189, N19 |
| Atrial fibrillation | 427.3 | I480, I481, I482, I483, I484, I4891, I4892 |
| Coronary artery disease | 413, 414, 429.2 | I201, I208, I209, I2510, I25110, I25111, I25118, I25119, I253, I2541, I2542, I255, I256, I25700, I25701, I25708, I25709, I25710, I25711, I25718, I25719, I25720, I25721, I25728, I25729, I25730, I25731, I25738, I25739, I25750, I25751, I25758, I25759, I25760, I25761, I25768, I25769, I25790, I25791, I25798, I25799, I25810, I25811, I25812, I2589, I259 |
| IS | 433,434,436 | I6300, I63011, I63012, I63019, I6302, I63031, I63032, I63039, I6309, I6310, I63111, I63112, I63119, I6312, I63131, I63132, I63139, I6319, I6320, I63211, I63212, I63219, I6322, I63231, I63232, I63239, I6329, I6330, I63311, I63312, I63319, I63321, I63322, I63329, I63331, I63332, I63339, I63341, I63342, I63349, I6339, I6340, I63411, I63412, I63419, I63421, I63422, I63429, I63431, I63432, I63439, I63441, I63442, I63449, I6349, I6350, I63511, I63512, I63519, I63521, I63522, I63529, I63531, I63532, I63539, I63541, I63542, I63549, I6359, I636, I638, I639, I6501, I6502, I6503, I6509, I651, I6521, I6522, I6523, I6529, I658, I659, I6601, I6602, I6603, I6609, I6611, I6612, I6613, I6619, I6621, I6622, I6623, I6629, I663, I668, I669, I6789 |
| AMI | 410 | I2101, I2102, I2109, I2111, I2119, I2121, I2129, I213, I214, I220, I221, I222, I228, I229 |
| Glaucoma | 365.1 | H4010X0, H4010X1, H4010X2, H4010X3, H4010X4, H4011X0, H4011X1, H4011X2, H4011X3, H4011X4, H401210, H401211, H401212, H401213, H401214, H401220, H401221, H401222, H401223, H401224, H401230, H401231, H401232, H401233, H401234, H401290, H401291, H401292, H401293, H401294, H401310, H401311, H401312, H401313, H401314, H401320, H401321, H401322, H401323, H401324, H401330, H401331, H401332, H401333, H401334, H401390, H401391, H401392, H401393, H401394, H40151, H40152, H40153, H40159, Q150 |
| Ranibizumab | S01LA04 |  |
| Aflibercept | S01LA05 |  |

nAMD= neovascular age-related macular degeneration; AMI= acute myocardial infraction; ATC= Anatomical Therapeutic Chemical; RVO = retinal vein occlusion; ICD-9-CM= International Classification of Diseases, Ninth Revision, Clinical Modification; IS = ischemic stroke
