# Supplementary material for: Comparison of risks of arterial thromboembolic events and glaucoma with ranibizumab and aflibercept intravitreous injection: A nationwide population‐based cohort study
Source: PLoS One. 2022 Apr 18;17(4):e0267088. doi: 10.1371/journal.pone.0267088 (PMC9015139; doi:10.1371/journal.pone.0267088)
Supplement: S2 Table — (DOCX) [file pone.0267088.s002.docx]

**S2 Table. Incidence (per 100 PY) and Adjusted HR of ATEs among retinal disease subgroups within 2-Year Follow-up Periods**

|  | Treatment | No. of ATEs | PY | Incidence(95% CI) | Adjusted*HR (95% CI) | *P* |
| --- | --- | --- | --- | --- | --- | --- |
| **nAMD** | **IVR** | 169 | 13,584 | 12.44 (10.70-14.47) | 1.00 (Ref.) |  |
|  | **IVA** | 24 | 2,808 | 8.50 (5.48-12.29) | 0.68 (0.47-1.00) | .048 |
| **DME** | **IVR** | 207 | 8,034 | 25.75 (22.37-29.39) | 1.00 (Ref.) |  |
|  | **IVA** | 42 | 1,495 | 28.17 (20.81-37.97) | 1.04 (0.48-2.25) | .916 |
| **RVO** | **IVR** | 5 | 212 | 21.66 (7.67-48.39) | 1.00 (Ref.) |  |
|  | **IVA** | 2 | 63 | 31.61 (3.86-88.77) | 1.27 (0.15-10.7) | .828 |
| **Others** | **IVR** | 71 | 6,619 | 10.76 (8.51-13.53) | 1.00 (Ref.) |  |
|  | **IVA** | 14 | 1,538 | 8.95 (4.98-14.45) | 0.85 (0.52-1.39) | .520 |

Adjusted HR was calculated using Cox proportional hazard analysis adjusted for all variables listed in Table 1.

Abbreviations: ATE = arterial thromboembolic event; HR = hazard ratio; nAMD=neovascular age-related macular degeneration; DME=diabetic macular edema ; CRVO=central retina vein occlusion; IVA = intravitreal aflibercept; IVR = intravitreal ranibizumab; PY = person-years.; CI = confidence interval; Ref.=reference
