# Supplementary material for: Comparison of risks of arterial thromboembolic events and glaucoma with ranibizumab and aflibercept intravitreous injection: A nationwide population‐based cohort study
Source: PLoS One. 2022 Apr 18;17(4):e0267088. doi: 10.1371/journal.pone.0267088 (PMC9015139; doi:10.1371/journal.pone.0267088)
Supplement: Attachment — Submitted filename: renamed_7ae72.docx [file pone.0267088.s003.docx]

Reviewer #1:

1. Very nicely written manuscript and is an important information.

Response: Thank you very much.

2. It would be good to analyse the retinal disease subgroups, Vein occlusion Vs AMD Vs DME, as some of these have associated systemic diseases more prone for thromboembolic risk.

**Response:** Thank you for your suggestion. As you mentioned, some of these disease has been shown associated with systemic diseases, especially DME and CRVO. According to our analysis (S2 Table), we found that the risk of ATE after anti-VEGF therapy was higher in the DME group and RVO group. In the revised manuscript, we provided the analysis to show the risk of ATE in the different retinal disease groups (S2 Table) and also added the discussion of the risk for ATE in different disease groups as your suggestion. (Page 18, Discussion Section, line 294- 299)

| S2 Table. Incidence (Per 100 PY) and adjusted HR of ATE among different retinal disease groups within two-year follow-up periods | | | | | | |
| --- | --- | --- | --- | --- | --- | --- |
| **Retinal disease groups** | **Treatment** | **No. of**  **ATE** | **PY** | **Incidence**  **(95% CI)** | **Adjusted*HR**  **(95% CI)** | ***P*** |
| nAMD | **IVR** | 169 | 13,584 | 1.24 (1.07-1.45) | 1.00 (Ref.) |  |
|  | **IVA** | 24 | 2,808 | 8.5 (0.55-1.23) | 0.68 (0.47-1.00) | 0.048 |
| DME | **IVR** | 207 | 8,034 | 2.58 (2.24-2.94) | 1.00 (Ref.) |  |
|  | **IVA** | 42 | 1,495 | 2.82 (2.08-3.80) | 1.04 (0.48-2.25) | 0.916 |
| RVO | **IVR** | 5 | 212 | 2.17 (0.77-4.84) | 1.00 (Ref.) |  |
|  | **IVA** | 2 | 63 | 3.16 (0.39-8.88) | 1.27 (0.15-10.7) | 0.828 |
| Others | **IVR** | 71 | 6,619 | 1.08 (0.85-1.35) | 1.00 (Ref.) |  |
|  | **IVA** | 14 | 1,538 | 0.90 (0.50-1.45) | 0.85 (0.52-1.39) | 0.520 |

*Adjusted HR was performed by Cox proportional hazard adjusted for all variables listed in Table 1.

Abbreviation: ATE = acute thromboembolic event; DME= Diabetic maculopathy; HR = hazard ratio; IVR = intravitreal ranibizumab; IVA = intravitreal aflibercept; PY=person year; Ref.=reference

3. There are inherent flaws due to the retrospective design, however, as it is nationwide data, it outweighs this. More details on the source of data would add value.

**Response:** Thank you. We provided more details on the source of data in the revised manuscript as your suggestion (Page 7, Materials and methods section, line 127-142):

Baseline characteristics of patients were derived from the National Health Insurance Research Data (NHIRD), a reimbursement claims database that covers 99% of the residents in Taiwan enrolled in the National Health Insurance (NHI) program. NHI is a single-payer health insurance system and has a contract with most healthcare providers in Taiwan. NHI provides a universal coverage that covers all necessary medical expenses including outpatient visits, the inpatient system, prescriptions, treatment with traditional Chinese medicine, dental services, operations, and investigations such as X-rays or magnetic resonance imaging. It is mandatory for care providers to upload the claims data from each service to the National Health Insurance Administration. Therefore, the NHIRD files include inpatient, outpatient, and pharmaceutical reimbursement claims, and the disease diagnosis was coded using the International Classification of Diseases, 9th Revision, Clinical Modification (ICD-9-CM) and 10th revision (ICD-10-CM) codes after the year 2016. Because patient data are deidentified in the NHIRD before release to researchers, the requirement for informed consent under the full review process of the TMU-JIRB was waived.

Reviewer #2: This is an important study and has been well executed and written. Following are some suggestions which should be taken into account while revising the manuscript.

MAJOR COMMENTS

4. The data of thromboembolic adverse events of aflibercept and ranibizumab from the protocol T of [drcr.net](http://drcr.net/) should be mentioned in the Introduction and Discussion section.

**Response:**

Thank you for your suggestion. We had added the data from the protocol T of DRCR.net in the Introduction (Page 6, line 101-112) and Discussion (Page 18, line 292 -302).

MINOR COMMENTS

5. Title should be changed to following: Comparison of risks of Arterial Thromboembolic Events and Glaucoma with Ranibizumab and Aflibercept Intravitreous Injection: A Nationwide Population‐Based Cohort Study

**Response:** Thanks for your suggestion. We changed the title as your suggestion. Please check the title in the revised manuscript.

6. Line 137: “missing sex information” in the exclusion criteria should be changed to “missing gender data”

**Response:** Thanks for your suggestion. We changed the missing sex information into missing gender information. Please check the revised manuscript (Fig 1, line 5).

7. What does the hyperlink in Line 152 refer to?

**Response:** We provided the references for the description of the CHA2DS2score. (Page 26 , line 431-433, Reference 17 )

8. The section of strength and limitations should be shortened.

**Response:** Thanks for your suggestion. We have shorten the strength and limitation section in the revised manuscript your suggestion.

(Page 21 , line 334)

4.4 Strengths and Limitations. The study patients was collected from a single-payer health insurance claim data that can reduce the underpowered study bias when evaluating rare adverse events. Moreover, the study cohort included patients who are usually excluded from clinical trials which represents real-world circumstances. Finally, we used strict criteria to select our sample that increase validity of the findings. This study has some limitations. First, the population data were derived from Taiwan, and hence, the results might not be generalizable to other ethnic groups. Second, the study was designed retrospectively, so caution should be exercised in drawing inferences. Third, limited subgroup analyses could be performed because the claims database did not include the records of visual acuity, anatomical features, optical coherence tomography, or intravenous fluorescein angiography results. Also, the diagnoses of retinal disease might not have been completely identified because the NHIRD only provided the data of the first 3 diagnoses for each outpatient claim. Finally, IPTW is increasingly used to estimate the effects of exposures using observational data; however, IPTW could not control unknown confounding factors.
