# Supplementary material for: Comparison of risks of arterial thromboembolic events and glaucoma with ranibizumab and aflibercept intravitreous injection: A nationwide population‐based cohort study
Source: PLoS One. 2022 Apr 18;17(4):e0267088. doi: 10.1371/journal.pone.0267088 (PMC9015139; doi:10.1371/journal.pone.0267088)
Supplement: Attachment — Submitted filename: Rebuttal letter0403.docx [file pone.0267088.s004.docx]

Thank you for the editors and the reviewers’ comments. We revised the manuscript as your suggestion:

1. Please review your reference list to ensure that it is complete and correct.

Response: The references of manuscript had been revised as follows:

- Reference 6 changed to Chou Y-B, Chen M-J, Lin T-C, Chen S-J, Hwang D-K. Priority options of anti-vascular endothelial growth factor agents in wet age-related macular degeneration under the National Health Insurance Program. Journal of the Chinese Medical Association. 2019;82(8), which is relevant to the content.(Page 25, line375, Reference 6)
- Some errors were noted on the Reference 11,12,13,14,15,16 and 17. We had revised them. Thank you for your valuable suggestion.

(Page 7, line 105, line 111; Page 8, line 131;Page 10, line 159, line 169)

- Reference 30 changed to Ahn J, Jang K, Sohn J, Park JI, Hwang DD-J. Effect of intravitreal ranibizumab and aflibercept injections on retinal nerve fiber layer thickness. Scientific Reports. 2021;11(1):5010. doi: 10.1038/s41598-021-84648-1 (Page 29, line 454, Reference 30)

2. If you need to cite a retracted article, indicate the article’s retracted status in the References list and also include a citation and full reference for the retraction notice.

Response: All references have been checked with the retraction database, and no retracted reference article was identified.

3. We corrected the name of first name to Yin-Hsi Chang.(Page 1,line 5)
4. We added the description of the abbreviation “FDA” (Page 5, line 77)
