# Supplementary material for: Comparison of risks of arterial thromboembolic events and glaucoma with ranibizumab and aflibercept intravitreous injection: A nationwide population‐based cohort study
Source: PLoS One. 2022 Apr 18;17(4):e0267088. doi: 10.1371/journal.pone.0267088 (PMC9015139; doi:10.1371/journal.pone.0267088)
Supplement: Attachment — Submitted filename: Major comments_20211231.docx [file pone.0267088.s006.docx]

**Major comments.**

1. **In the introduction: “However, few studies have compared the risk of elevated IOP between ranibizumab and aflibercept’. Authors need to quote the studies which they mention in this sentence.**

**Reply:** Thanks for your comments. We have added the references to the following sentence in the revised manuscript : “However, few studies have compared the risk of elevated IOP between ranibizumab and aflibercept [15-17] ”(page 7, line 115-116 ). We also revised the list of references(page 27-28 , line 426-436)

2. **Why do authors think that comparing the intraocular pressure rise between ranibizumab and aflibercept was justified? Why do they think that the rise would be different between two drugs?**

**Reply:** Thanks the valuable comment.

In this study ,we used three claims of diagnosis codes with anti-glaucoma medication to increase the validity of diagnosis. (page 11 , line 201-204).One of the limitations of this study is that we did not get the detail records of IOP, and we only used the diagnosis codes with glaucoma medication prescription to compare the risks of glaucoma(page 23, line 356-357).

Several studies have reported IOP elevation after intravitreal injection,^1-3^ and the repeated elevations in IOP after intravitreal injections could be correlated with an increased incidence of glaucoma.^4,5,6^ The risks of glaucoma is concerned because patients with chronic retinal diseases may require multiple intravitreal injections over time. The injection procedure itself does not vary considerably between different anti-VEGF agents^7^; therefore, we postulated that the agent itself rather than the injection procedure is related to the observed elevations in IOP. Limited numbers of studies have compared the risks of glaucoma between different anti-VEGF agents.^7^ Therefore, we compared the risks of glaucoma between these two agents.We have added more discussions to the revised manuscript(page 21-22 , line 321-345).

References:

1.Good TJ, Kimura AE, Mandava N, Kahook MY. Sustained elevation of intraocular pressure after intravitreal injections of anti-VEGF agents. *The British journal of ophthalmology.* 2011;95(8):1111-1114.

2.Tseng JJ, Vance SK, Della Torre KE, Mendonca LS, Cooney MJ, Klancnik JM, Sorenson JA, Freund KB. Sustained increased intraocular pressure related to intravitreal antivascular endothelial growth factor therapy for neovascular age-related macular degeneration. *Journal of glaucoma.* 2012;21(4):241-247.

3. Bracha P, Moore NA, Ciulla TA, WuDunn D, Cantor LB. The acute and chronic effects of intravitreal anti-vascular endothelial growth factor injections on intraocular pressure: A review. *Survey of ophthalmology.* 2018;63(3):281-295.

4. de Vries VA, Bassil FL, Ramdas WD. The effects of intravitreal injections on intraocular pressure and retinal nerve fiber layer: a systematic review and meta-analysis. *Sci Rep*. 2020;10(1):13248. Published 2020 Aug 6. doi:10.1038/s41598-020-70269-7

5. Du, J. , Patrie, J. T. , Prum, B. E. , Netland, P. A. & Shildkrot, Y. (. (2019). Effects of Intravitreal Anti-VEGF Therapy on Glaucoma-like Progression in Susceptible Eyes. *Journal of Glaucoma, 28* (12), 1035-1040. doi: 10.1097/IJG.0000000000001382.

6. Mansoori T, Agraharam SG, Manwani S, Balakrishna N. Intraocular Pressure Changes after Intravitreal Bevacizumab or Ranibizumab Injection: A Retrospective Study. *J Curr Ophthalmol*. 2021;33(1):6-11. Published 2021 Mar 26. doi:10.4103/JOCO.JOCO_5_20

7. Ramsey, D.J., McCullum, J.C., Steinberger, E.E. *et al.* Intraocular pressure decreases in eyes with glaucoma-related diagnoses after conversion to aflibercept for treatment-resistant age-related macular degeneration. *Eye* (2021). https://doi.org/10.1038/s41433-021-01729-1

3. **In the result: “In patients without a history of glaucoma, the glaucoma risk was not significantly different between the IVA group and the IVR group (adjusted HR: 0.63, 95% CI: 0.37–1.06, P 239 = .084; Table 3). The criteria to define glaucoma have not been mentioned in the Method section.**

**Reply:** Thanks for your comments.

In the manuscript, we described the the definition of glaucoma in the Method-Main Outcome Measurements sections (page 11, line 200-202): “Glaucoma was defined as at least three times of diagnoses based on ICD-9-CM or ICD-10-CM diagnostic codes with antiglaucoma agents (ATC code S01E) administration”.The details related to the dignositc codes are provided in the Supporting Information section (S1 Table). In addition, we have added additional relevant information in the Method section.(page 11, line 203-204).

4. **This is the single sentence in the Results which deals with intraocular pressure rise aspect in the study. Intraocular pressure monitoring is standard care before and after** **intravitreal injection. Why do authors chose to report and combine intraocular pressure data along side systemic thromboembolic adverse events as both are completely unconnected.**

**Reply:** Thanks for your valuable comments.

We added additional information about glaucoma issue in the Discussion section(page 21, line 321-344).

Patients with chronic retinal diseases require multiple injections; therefore, the risks of systemic and ocular adverse events constitute a concern.The major systemic adverse events associated with the injection of anti-VEGF agents are arterial thromboembolic events(ATEs) , and the risks of ATEs between the two drugs are concerned due to many patients receiving intravitreal injection are high risks for ATEs , such as old aged patients and DM patients. In addition, frequently reported ocular adverse effects with anti-VEGF intravitreal injection include the cataract, glaucoma, and conjunctival hemorrhage.^1,2^ However, the increased risks of glaucoma are concerning due to the irreversible optic neuropathy that may result.Therefore, we chose glaucoma as an indicator of these ocular adverse events.

Because the injection procedure itself does not differ between two drugs, the medication itself may play a role in the systemic and ocular adverse events. Aflibercept had a longer half-life than ranibizumab, and inflammation appears more common in aflibercept than in ranibizumab.^3^ The inflammation could affect aqueous humor production or outflow pathways, such as the trabecular meshwork.^4^Previous studies had demonstrated that elevated inflammatory cytokines in POAG patients,^5,6^and chronic inflammation plays an important role in glaucoma mechanism.^7^Inflammation also plays a critical role in all stages of atherosclerotic plaques formation, leading to the acute coronary and cerebrovascular syndromes.^8^We discussed the issue about inflammation of these drugs in the revised manuscript(page 20, line 311-312, and page22, line 331-333).Addtional studies will be need to evaluate the systemic and intraocular inflammation reaction between these two drugs, and the link between the inflammation and the risks of those systemic and ocular adverse events.

References:

1. Holz FG, Figueroa MS, Bandello F, et al. RANIBIZUMAB TREATMENT IN TREATMENT-NAIVE NEOVASCULAR AGE-RELATED MACULAR DEGENERATION: Results From LUMINOUS, a Global Real-World Study. *Retina*. 2020;40(9):1673-1685. doi:10.1097/IAE.0000000000002670
2. Scott IU, VanVeldhuisen PC, Ip MS, Blodi BA, Oden NL, Awh CC, Kunimoto DY, Marcus DM, Wroblewski JJ, King J; SCORE2 Investigator Group. Effect of Bevacizumab vs Aflibercept on Visual Acuity Among Patients With Macular Edema Due to Central Retinal Vein Occlusion: The SCORE2 Randomized Clinical Trial. JAMA. 2017 May 23;317(20):2072-2087. doi: 10.1001/jama.2017.4568. PMID: 28492910; PMCID: PMC5710547.
3. Souied EH, Dugel PU, Ferreira A, Hashmonay R, Lu J, Kelly SP. Severe Ocular Inflammation Following Ranibizumab or Aflibercept Injections for Age-Related Macular Degeneration: A Retrospective Claims Database Analysis. *Ophthalmic Epidemiol*. 2016;23(2):71-79. doi:10.3109/09286586.2015.1090004
4. Ramsey, D.J., McCullum, J.C., Steinberger, E.E. *et al.* Intraocular pressure decreases in eyes with glaucoma-related diagnoses after conversion to aflibercept for treatment-resistant age-related macular degeneration. *Eye* (2021). <https://doi.org/10.1038/s41433-021-01729-1>
5. Ten Berge JC, Fazil Z, van den Born I, et al. Intraocular cytokine profile and autoimmune reactions in retinitis pigmentosa, age-related macular degeneration, glaucoma and cataract. *Acta Ophthalmol*. 2019;97(2):185-192. doi:10.1111/aos.13899
6. Burgos-Blasco B, Vidal-Villegas B, Saenz-Frances F, Morales-Fernandez L, Perucho-Gonzalez L, Garcia-Feijoo J, Martinez-de-la-Casa JM. Tear and aqueous humour cytokine profile in primary open-angle glaucoma. Acta Ophthalmol. 2020 Sep;98(6):e768-e772. doi: 10.1111/aos.14374. Epub 2020 Feb 11. PMID: 32043817.
7. Baudouin C, Kolko M, Melik-Parsadaniantz S, Messmer EM. Inflammation in Glaucoma: From the back to the front of the eye, and beyond. Prog Retin Eye Res. 2021 Jul;83:100916. doi: 10.1016/j.preteyeres.2020.100916. Epub 2020 Oct 17. PMID: 33075485.
8. Arnott C, Punnia-Moorthy G, Tan J, Sadeghipour S, Bursill C, Patel S. The Vascular Endothelial Growth Factor Inhibitors Ranibizumab and Aflibercept Markedly Increase Expression of Atherosclerosis-Associated Inflammatory Mediators on Vascular Endothelial Cells. *PLoS One*. 2016;11(3):e0150688. Published 2016 Mar 9. doi:10.1371/journal.pone.0150688

**Minor comments.**

9. **In the Methods: “Because** **patients data are deidenetifed in the NHIRD before release to researchers, the requirement for informed consent under the full review process of the TMU-JIRB was waive.” Does the data storage policy include patients’ consent permitting use of their deidentifed data for research purpose? ‘Additionally, the study used the National Death Registry under the regulation of Health and Welfare Data Science Center, Ministry of Health and Welfare, Executive Yuan, Taiwan.’ The use of this data in the study needs to be elaborated.**

**Response:** Thanks for your comments. Because the study involved data derived from human subjects, patients’ privacy and data confidentiality are major concern. To protect the privacy of patients, patient data in the NHIRD were deidentified before they are released to researchers. The need for informed consent was waived because it was impossible to obtain from patients. According to the data agreement regulations, the use of the data must be for research purposes only. All applications are to be reviewed by peer experts to ensure rational data use. In addition, researchers must follow Taiwan’s Computer-Processed Personal Data Protection Law and related regulations and sign an agreement declaring that no attempt will be made to retrieve information that would potentially violate the privacy of patients or health-care providers.^1^ The NHIRD encrypts personal patient information to protect privacy and provides researchers with anonymous identification numbers associated with relevant claims information^1^, including sex, date of birth, medical services received, and prescriptions received. Therefore,patient consent is not required to access the NHIRD, and as such, the Joint Institutional Review Board of Taipei Medical University issued a formal written waiver to obviate the need for informed consent. This study was approved as fulfilling those conditions required for exemption by the Institutional Review Board of Taipei Medical University (TMU-JIRB No.202005104).We have added these information to the revised manuscript(page 7, line 125-130).

Moreover, the two major databases used in the study, namely the NHIRD and the National Death Registry, can be linked through a unique deidentifier. To protect personal information, researchers are required to perform their analyses on the servers provided by the Health and Welfare Data Center (HWDC), Ministry of Health and Welfare (MOHW), Taiwan. According to this regulation, no individual-level data can be taken out, and all results must be reviewed by data custodians to prevent any disclosure of patient identity.We have added additional relevant information to the revised manuscript(page 8, line145-153).

References

1. Hsieh CY, Su CC, Shao SC, et al. Taiwan's National Health Insurance Research Database: past and future. *Clin Epidemiol*. 2019;11:349-358. Published 2019 May 3. doi:10.2147/CLEP.S196293

.

10. **In Discussion section: “****Besides, the Singpore study was performed in a single cener; therefore, the data did not represent the national population”. Do authors really think that this criticism was valid in view of small size and population of city state Singapore.**

**Reply:** Thank you for your suggestions.We agreed that the criticism is inapporiate, and have deleted the sentence (page 18 , line 275).

11. **In Discussion section:** **“The present study showed the risk of ATEs in DME patients did not significantly differ between the IVA and IVR groups, but we did not specifically compare the risks of ATEs in DME patients with a history of such events.” The second part of this sentence is unclear.**

**Reply:** Thank you for your suggestion. We revised the sentence “The present study showed the risk of ATEs in DME patients did not significantly differ between the IVA and IVR groups, but we did not specifically compare the risks of ATEs in DME patients with a history of APTC events.” (page 20 , line 300-301).

12. **The section of Discussion dealing with “Risk of Glaucoma” appears nondescript and not clearly written. It does not drive home the intended message.**

**Reply:** Thanks for your comments, and we revised the Discussion section “Risk of Glaucoma” (page 21-22 , line 321-344).

.
