# Supplementary material for: Comparison of risks of arterial thromboembolic events and glaucoma with ranibizumab and aflibercept intravitreous injection: A nationwide population‐based cohort study
Source: PLoS One. 2022 Apr 18;17(4):e0267088. doi: 10.1371/journal.pone.0267088 (PMC9015139; doi:10.1371/journal.pone.0267088)
Supplement: Attachment — Submitted filename: Major comments_20220218.docx [file pone.0267088.s007.docx]

Reviewers' comments:

1. Have the authors made all data underlying the findings in their manuscript fully available? The [PLOS Data policy](http://www.plosone.org/static/policies.action#sharing) requires authors to make all data underlying the findings described in their manuscript fully available without restriction, with rare exception (please refer to the Data Availability Statement in the manuscript PDF file). The data should be provided as part of the manuscript or its supporting information, or deposited to a public repository. For example, in addition to summary statistics, the data points behind means, medians and variance measures should be available. If there are restrictions on publicly sharing data—e.g. participant privacy or use of data from a third party—those must be specified.

**Reply:** We have added the Data Availability statement in the revised manuscript (page 24, line 377-381).

2. **Reviewer 4:** Definition of glaucoma used to identify cases needs to be provided in the Methods section. It needs to be discretely mentioned.

**Reply:** Thanks the valuable comment. We have added more description about the definition of glaucoma cases in the revised manuscript (page 11, line 201-208).
