# Supplementary material for: Comparison of risks of arterial thromboembolic events and glaucoma with ranibizumab and aflibercept intravitreous injection: A nationwide population‐based cohort study
Source: PLoS One. 2022 Apr 18;17(4):e0267088. doi: 10.1371/journal.pone.0267088 (PMC9015139; doi:10.1371/journal.pone.0267088)
Supplement: Attachment — Submitted filename: Plos one reviewer comment 0806.docx [file pone.0267088.s005.docx]

- **For Journal requirement:**

Reply:

1. We had sent the manuscript to a professional English editing service to revise all the typographical or grammatical errors.
2. We have verified all the references on the website Retraction Watch.
3. Reference 30 changed to Ahn J, Jang K, Sohn J, Park JI, Hwang DD-J. Effect of intravitreal ranibizumab and aflibercept injections on retinal nerve fiber layer thickness. Scientific Reports. 2021;11(1):5010. doi: 10.1038/s41598-021-84648-1

(page 22, line 331-332;page 29, line 463, reference 30)

- **For reviewer #2**: Thank you for your comments.
- **For reviewer #3**

1. **Page 11, line 78: FDA approval is given for a particular disease, for eg, Ranibizumab was approved for treating wet AMD in 2006, whereas Aflibercept was approved in 2011 for the same disease. Are you pointing out that Taiwan FDA approved these two drugs for all the diseases mentioned in the study together? Please modify your statements here.**

Reply: Thank you for your comments.

1. Similar to the United States, the use of anti-VEGF agents was first approved for nAMD treatment in Taiwan. Ranibizumab and aflibercept were approved for treating nAMD by the Taiwan FDA in 2009 and 2011, respectively. Their use for other indications including DME was approved in subsequent years. Currently, these agents can be used in the treatment of nAMD, DME, CRVO, BRVO, PCV and myopic CNV. We have made revisions as follow:

Ranibizumab as a treatment for retinal neovascularization diseases such as neovascular age-related macular degeneration (nAMD) was approved for use by the US Food and Drug administration (FDA) in June 2006 and by the Taiwan FDA since 2009. Aflibercept (Eylea, Regeneron, Tarrytown, PA, USA and Bayer HealthCare, Berlin, Germany) was also approved by the US FDA in November 2011 and by the Taiwan FDA in June 2013 for treating nAMD, and its use has rapidly increased since then. (page 5, line 75-81)

**2. There are gross Grammatical errors, please check editing as well.**
Reply: We have availed a professional English editing service to recheck the manuscript and revise all the grammatical errors of the manuscript.

page 3: line 41, 43: add “the”, line 50 add “those”

page 4: line 52: add “the”

line 54: “was not significantly different” change to “did not significantly differ”

line 57:” our study suggested that that these two intravitreal agents had no significant difference ” change to “No significant differences in the risk of major ATEs and glaucoma were found between ranibizumab and aflibercept,”

page 5: line 83 add “that of”

page 6: line 87: add “related to the use of these agents”

line 95: add “who received”

line98: add “in the treatment of”

line100: “ATPC” change to “APTC”

page 7: line 109: “quickly” change to “rapidly”

line 109: add “and”

page 8: line125: add “The” “program”

line126: add “that”

line128: add “s”

page 9: line 135: add “The”

line 145: “consisted of“ change to “comprised”

page 13: line 206: add “occurred”

line 209: add “The”

line 211: add “The”

line 215: add “the”

line 217 and line 218: add “USA”

page 14: line 232,234,235: add “the”

page 18; line 264: add “s”

line 266: “these two anti-VEGF agents exhibited no difference in terms of the aforementioned side effects.” change to “the aforementioned side effects did not differ between the two anti-VEGF agents”

page 19; line 280: add “cohort”

line: 284: add “the”

page 20: line 296. add “the”

line 298: add “s”

line 301. add “the”

line 306: add “the”

page 21:line320:add “the”

line 322, 324: add “the”

line 327: add “an”

Thank you for your suggestion.

**3. Please explain: ICDR-9 and 10 CM coding was acceptable after 2016, whereas you selected the patients between 2011 and 2016. Does that mean that the ICDR coding was modified after 2016 for the patients seen before that year? Or there is a discrepancy in diagnosis and selection bias here because of the different ICDR coding definitions?**

Reply: Thank you for your suggestion,

1. Because the prescriptions of ranibizumab and aflibercept were first reimbursed by the National Health Insurance in Taiwan on January 1, 2011, and August 1, 2014, respectively, we selected a study period of 2011–2016. We selected 2017 as the follow-up year to ensure that at least 1 year of follow-up data were available for all eligible patients (page 9, line 148–150).
2. The ICDR coding was modified after 2016 for the patients seen before that year, and there might be a discrepancy in diagnosis because of the different ICDR coding. However, we separated the study patients into two groups based on the prescription claims, not based on the diagnostic codes. Because we used the same methods and medical coding systems to compare the outcomes between the IVR and IVA groups, no selection bias was noted between the two groups.
3. We have presented all the ICDR codes in Table S1. Care providers must upload the medical records of patients’ reimbursement applications for ranibizumab and aflibercept. Approval for ranibizumab and aflibercept reimbursement is not based on medical coding alone but also includes a review of the patient’s medical records by a retinal specialist. The review process increases the validity of the diagnosis. We have added the relevant explanation in the revised manuscript.(page 8, line 131–134)

**4. Please include the following in exclusion/inclusion criteria: patients with poor systemic profile, and what was the minimum interval between the last episode of cardiovascular event and injection AntiVEGF?**

Reply: Thank you for your valuable comments.

1. In our study, we used inverse probability treatment weighting based on the propensity score to balance the baseline characteristics between the two groups. Although we did not specifically include or exclude patients with poor systemic profiles, we did separately analyze the risk in patients with and without a history of AMI or stroke. (page 17. Table 2)
2. Patients with poor systemic profiles might be excluded from the treatment because of the high risks of systemic adverse effects. However, this claim-based data study was based on the disease diagnostic codes and prescription claims. The detailed information of exclusion criteria for each doctor is not provided in this claims database. We have added the relevant explanation in the revised manuscript (page 23, line 350-352) However, we believe that this limitation might not bias our results because we used the same methods to compare the outcomes between the two groups.
3. We separately analyzed the data in patients with and without a history of AMI or stroke, and we did not specifically exclude patients with recent cardiovascular event. The definition of cardiovascular events was based on the diagnostic codes and hospitalization (page12, line 187-190). A history of AMI or stroke was defined as the occurrence of AMI or stroke events before the index date (anti-VEGF injection). We did not specifically record the last episode of cardiovascular events of each patient, and a further study for patients with recurrent stroke or AMI events after intravitreal injection will be needed in the future.

**4 Please explain this: You have excluded patients below 50 years of age. Yet, for the Inverse Probability Treatment Weighting, you have used CHA2DS2 scoring system which scores patients above 65 and above 75 years. This puts the patients between 50-65 years as confounded due to age, as they could not be scored for weight adjustments.**

Reply: Thank you for your valuable comments.

1. We excluded patients below 50 years of age because the risk of ATE is low among patients younger than 50 years old. Because both the IVR and IVA groups had the same exclusion criteria, the exclusion of patients younger than 50 years should not have biased our results, although generalizability to young patients was limited. We have added the relevant explanation in the revised manuscript (page 22, line 342-344)
2. We used the CHA2DS2 scoring system as a covariate to adjust for its confounding effect on cardiovascular risk. According to the scoring system, a patient aged between 50 and 64 years is given 0 points. Thus, their scores were included for weight adjustments in the study. We have added this information in the revised manuscript.(page 11,line 170 )
   **6. To exclude the patients who are at increased risk of Glaucoma, the exclusion criteria must exclude pre-existing glaucoma/axial length variations/ phakic status/ patients status post laser/ vitrectomy and steroid use. These are important to differentiate between patients who developed glaucoma due to a particular AntiVEGF agent versus those who had a high tendency to develop glaucoma after any injection.**

Reply: Thanks for your comments.

1. Several studies had reported preexisting glaucoma to be a risk factor for sustained ocular hypertension after AntiVEGF injection^1,2^.

In our comparison of the risk of glaucoma between the two groups, we excluded	patients with preexisting glaucoma and only compared patients without a history	of glaucoma on Table 3. (page 14and 15, line 236–240, and page 17, Table 3) No significant differences in glaucoma development were noted between the two groups.

2. This claim-based study was based on the claims data of diagnostic codes, prescriptions, and procedures. However, the database did not include the data on axial length, anatomical features, or OCT results. We have described this as a limitation of the study in the revised manuscript.(page 22 and 23 ,line 345-347)

References:

1. Maruyama-Inoue, M., Inoue, T., Mohamed, S. *et al.* Incidence of elevated intraocular pressure after intravitreal injection in Japanese patients with age-related macular degeneration. *Sci Rep* **11,** 12246 (2021). https://doi.org/10.1038/s41598-021-91832-w

2. Dedania VS, Bakri SJ. SUSTAINED ELEVATION OF INTRAOCULAR PRESSURE AFTER INTRAVITREAL ANTI-VEGF AGENTS: What Is the Evidence? Retina. 2015 May;35(5):841-58. doi: 10.1097/IAE.0000000000000520. PMID: 25905784.

**7. You have included the incidence of adverse events occurring even after 2 years following the last AntiVEGF agent use. This would be inappropriate as the adverse event will definitely not reflect a direct cause and effect relationship with the AntiVEGF agent.**

Reply: Thank you for your comments,

1. Our patients were included on the date of their first anti-VEGF injection and followed up for up to 2 years. Although a direct causal effect could not be concluded from the present study, we provided long-term real-world data in terms of the cumulative risk.
2. FOCUS, MARINA, ANCHOR, PIER, and SAILOR were trials that assessed the outcomes of ranibizumab treatment at either 1 or 2 years.^1-5^ Bressler et al. pooled these studies to analyze the CVA risk at 1 year and 2 years.^6^ Kitchens et al. reviewed 10 phase II and III trials and calculated the risk of ocular and systemic adverse events as events per person-years (PYR) and rate ratios (RR) of aflibercept treatment.^7^ The study period of that also exceeded 1 year. Our study had a similar follow-up period.
3. An acute spike in IOP elevation after intravitreal injection has been reported in several studies, and even sustained IOP elevation after anti-VEGF injection has been revealed.^8-12^ However, a short term IOP spike after antiVEGF injection seems not associated with the damage to optic nerve^12^, and whether ocular hypertension develops into glaucoma requires repeat evaluations over the long term. For instance, Casa et al analyzed the retinal nerve fiber layer thickness changes in nAMD patients receiving anti-VEGF injection over 1 year.^13^ Filek et al found increasing cup-disc ratio over time in DME patients with anti-VEGF injection in a 2-year analysis.^14^ Our study was designed to compare the risk of glaucoma between the two groups, and the cases of glaucoma was defined as at least three times of diagnostic codes with antiglaucoma agents (ATC code S01E) claims.(page 12, line 190-191) Therefore, patients with short-term ocular hypertension will be excluded, and a follow-up period of 1-2 years is required to assess whether a significant risk of glaucoma exists.

1.	Rosenfeld PJ, Brown DM, Heier JS, Boyer DS, Kaiser PK, Chung CY, Kim RY. Ranibizumab for Neovascular Age-Related Macular Degeneration. *New England Journal of Medicine.* 2006;355(14):1419-1431.

2.	Regillo CD, Brown DM, Abraham P, Yue H, Ianchulev T, Schneider S, Shams N. Randomized, Double-Masked, Sham-Controlled Trial of Ranibizumab for Neovascular Age-related Macular Degeneration: PIER Study Year 1. *American journal of ophthalmology.* 2008;145(2):239-248.e235.

3.	Antoszyk AN, Tuomi L, Chung CY, Singh A. Ranibizumab combined with verteporfin photodynamic therapy in neovascular age-related macular degeneration (FOCUS): year 2 results. *American journal of ophthalmology.* 2008;145(5):862-874.

4.	Brown DM, Kaiser PK, Michels M, Soubrane G, Heier JS, Kim RY, Sy JP, Schneider S. Ranibizumab versus verteporfin for neovascular age-related macular degeneration. *The New England journal of medicine.* 2006;355(14):1432-1444.

5.	Brown DM, Michels M, Kaiser PK, Heier JS, Sy JP, Ianchulev T. Ranibizumab versus verteporfin photodynamic therapy for neovascular age-related macular degeneration: Two-year results of the ANCHOR study. *Ophthalmology.* 2009;116(1):57-65.e55.

6.	Bressler NM, Boyer DS, Williams DF, Butler S, Francom SF, Brown B, Di Nucci F, Cramm T, Tuomi LL, Ianchulev T, Rubio RG. Cerebrovascular accidents in patients treated for choroidal neovascularization with ranibizumab in randomized controlled trials. *Retina (Philadelphia, Pa).* 2012;32(9):1821-1828.

7.	Kitchens JW, Do DV, Boyer DS, Thompson D, Gibson A, Saroj N, Vitti R, Berliner AJ, Kaiser PK. Comprehensive Review of Ocular and Systemic Safety Events with Intravitreal Aflibercept Injection in Randomized Controlled Trials. *Ophthalmology.* 2016;123(7):1511-1520.

8.	Bressler SB, Almukhtar T, Bhorade A, Bressler NM, Glassman AR, Huang SS, Jampol LM, Kim JE, Melia M. Repeated intravitreous ranibizumab injections for diabetic macular edema and the risk of sustained elevation of intraocular pressure or the need for ocular hypotensive treatment. *JAMA ophthalmology.* 2015;133(5):589-597.

9.	Kim JE, Mantravadi AV, Hur EY, Covert DJ. Short-term Intraocular Pressure Changes Immediately After Intravitreal Injections of Anti–Vascular Endothelial Growth Factor Agents. *American journal of ophthalmology.* 2008;146(6):930-934.e931.

10.	Good TJ, Kimura AE, Mandava N, Kahook MY. Sustained elevation of intraocular pressure after intravitreal injections of anti-VEGF agents. *The British journal of ophthalmology.* 2011;95(8):1111-1114.

11.	Tseng JJ, Vance SK, Della Torre KE, Mendonca LS, Cooney MJ, Klancnik JM, Sorenson JA, Freund KB. Sustained increased intraocular pressure related to intravitreal antivascular endothelial growth factor therapy for neovascular age-related macular degeneration. *Journal of glaucoma.* 2012;21(4):241-247.

12.	Bracha P, Moore NA, Ciulla TA, WuDunn D, Cantor LB. The acute and chronic effects of intravitreal anti-vascular endothelial growth factor injections on intraocular pressure: A review. *Survey of ophthalmology.* 2018;63(3):281-295.

13.	Martinez-de-la-Casa JM, Ruiz-Calvo A, Saenz-Frances F, Reche-Frutos J, Calvo-Gonzalez C, Donate-Lopez J, Garcia-Feijoo J. Retinal nerve fiber layer thickness changes in patients with age-related macular degeneration treated with intravitreal ranibizumab. *Invest Ophthalmol Vis Sci.* 2012;53(10):6214-6218.

14.	Filek R, Hooper P, Sheidow TG, Gonder J, Chakrabarti S, Hutnik CM. Two-year analysis of changes in the optic nerve and retina following anti-VEGF treatments in diabetic macular edema patients. *Clinical ophthalmology (Auckland, NZ).* 2019;13:1087-1096.

8 **You have done an excellent job in S2 as the patients with DME and RVO should be analyzed separately from those with wet AMD, as the systemic profile of such patients may already be compromised to a great extent prior to AntiVEGF use.**

Reply: Thank you for your comments.

**9. In subgroup analysis in patients who developed glaucoma, please include the following: when was IOP measured after the injection, what was the IOP spike, how many AGMs were used to control the IOP and was the IOP controlled or not, when compared between the ranibizumab and aflibercept groups?**

Reply: Thank you for your comments.

1. In the manuscript, we have discussed this as a limitation of the claims-based study. The study was based on the claims data of diagnostic codes, prescriptions, and procedures. The database did not include the details of IOP, OCT, or visual field results.(page 22, line 345-347) Therefore, this database did not provide the information whether the IOP was controlled or not. Future work is needed to compare the risks of acute IOP elevation between these two agents, and assess the glaucoma control of these patients.
